# The Effect of Long-Term Cryopreservation on the Properties and Functionality of Platelet-Rich Plasma

**DOI:** 10.3390/ijms26020721

**Published:** 2025-01-16

**Authors:** Maider Beitia, Jorge Guadilla, Jon Mercader Ruiz, Daniel Marijuan Pinel, Pello Sánchez, Ane Iriondo, Renato Andrade, João Espregueira-Mendes, Diego Delgado, Mikel Sánchez

**Affiliations:** 1 Advanced Biological Therapy Unit, Hospital Vithas Vitoria, 01008 Vitoria-Gasteiz, Spain; maider.beitia@ucatrauma.com (M.B.); jon.mercader@ucatrauma.com (J.M.R.); daniel.marijuan@ucatrauma.com (D.M.P.); diego.delgado@ucatrauma.com (D.D.); 2 Arthroscopic Surgery Unit, Hospital Vithas Vitoria, 01008 Vitoria-Gasteiz, Spain; jorgue.guadilla@ucatrauma.com (J.G.); ane.iriondo@ucatrauma.com (A.I.); 3 Clínica Espregueira—FIFA Medical Centre of Excellence, 4350-415 Porto, Portugal; randrade@espregueira.com (R.A.); jem@espregueira.com (J.E.-M.); 4 Dom Henrique Research Centre, 4350-415 Porto, Portugal; 5 Porto Biomechanics Laboratory (LABIOMEP), Faculty of Sports, University of Porto, 4200-450 Porto, Portugal; 6 School of Medicine, University of Minho, 4710-057 Braga, Portugal; 7 ICVS/3B’s-PT Government Associate Laboratory, 4710-057 Braga, Portugal; 8 3B’s Research Group—Biomaterials, Biodegradables and Biomimetics, University of Minho, Headquarters of the European Institute of Excellence on Tissue Engineering and Regenerative Medicine, Barco, 4805-694 Guimarães, Portugal

**Keywords:** platelet-rich plasma, storage, cryopreservation, growth factors, bioactivity

## Abstract

Platelet-Rich Plasma (PRP) is a biological treatment widely used in regenerative medicine for its restorative capacity. Although PRP is typically applied at the time of obtention, long-term storage and preservation could enhance its versatility and clinical applications. The objective of this study was to evaluate the effect of long-term freezing on PRP. For that, PRP and Platelet Lysates (PL) were collected and preserved at −20 °C and −80 °C for 6 and 12 months. The parameters analyzed included platelet count and size, fibrinogen levels, platelet activation percentage, growth factor (GF) levels, and bioactivity on cultured dermal fibroblasts. No significant changes in platelet count were found; however, variations in platelet size were observed. Platelets stored at −20 °C and −80 °C showed structural changes and increased activation over time, including membrane roughness and possible aggregation. GF analysis revealed a reduction in platelet-derived growth factors (PDGF-AB and VEGF), while extraplatelet factors like IGF-1 remained stable. Fibroblast cultures showed comparable cell viability when exposed to fresh and cryopreserved PRP and PL samples. These findings suggest that cryopreserving PRP at −20 °C or −80 °C for up to 12 months is a feasible approach for retaining its therapeutic potential, supporting its use in biobanking, and expanding clinical accessibility.

## 1. Introduction

Over the last decade, molecular biology has revolutionized approaches to treatment in several medical specialties, offering new perspectives and tools for clinical practice [1,2]. Among these innovations, the use of Platelet Rich Plasma (PRP) stands out as an effective and promising therapy for its restorative, anti-inflammatory and analgesic properties [3,4,5]. PRP is a platelet concentrate usually obtained by the centrifugation of the patient’s own blood. Its therapeutic effect lies in the biological effects of the growth factors (GF) present in platelets, such us Transforming Growth Factor beta 1 (TGF-β1), Vascular Endothelial Growth Factor (VEGF) and Platelet-derived Growth Factor AB (PDGF-AB), which serve to accelerate the healing of damaged tissue. Moreover, they interact with plasmatic molecules, which are outside platelets and present in platelet lysates (PL) like Insulin-like Growth Factor 1 (IGF-1) or Hepatocyte Growth Factor (HGF) [5,6]. They stimulate tissue regeneration by promoting biological processes such as cell proliferation and migration, angiogenesis, and the inhibition of apoptosis, in addition to playing a part in modulating inflammation and fibrosis [7,8]. Thus, the mechanism of action of PRP is based on its biomolecular content, which is crucial for its bioactivity on the target tissue [9,10].

Nevertheless, the use of PRP in clinical practice has some limitations that may restrict a wider range of potential uses. Firstly, PRP is autologous in nature, and the entire process for its obtaining is typically carried out just before it is injected into the affected area. This means that the blood is collected and processed during the patient’s visit for each injection session, which can result in a longer waiting time for the patient. Secondly, the possibility of allogeneic use is restricted as both the patient and donor(s) would need to be scheduled to visit the medical center at the same time. Lastly, and as it is a treatment that often involves repeated injections, this process may pose a challenge for patients who have medical complications or anxiety regarding blood collection, as it requires blood extraction at every infiltration session.

Considering these drawbacks, PRP preservation strategies have become the focus of a number of studies. Although it has been determined that PRP can be preserved for a maximum of one week at room temperature (RT) without altering its biological activity [11,12,13], it is crucial to develop a preservation strategy that maintains the biochemical composition of PRP over longer periods. Freeze-drying has been proven to preserve the integrity of platelets and GFs for long periods of time in vitro [14] and in vivo [15], though clinical application could be challenging due to the use of specific equipment and higher costs. In this context, cryopreservation plays a crucial role as it allows the conservation of PRP, making it more accessible for clinical practice, as well as simpler and cheaper. Indeed, previous studies demonstrated that, although changes in the biochemical composition were observed, the cryopreservation of PRP maintains its biological activity in a period of 1 and 3 months both in vitro [16,17,18] and in vivo [19]. However, studies with a longer storage period are needed, since the study of the long-term cryopreservation of PRP would open up the possibility of developing biobanks. Thus, plasma could be stored to provide access to high-quality samples for long-term treatment, while also promoting research and the development of personalized treatments. This advance not only optimizes clinical outcomes but also opens new avenues for the understanding and management of traumatic injuries, marking a milestone in regenerative medicine.

As discussed in previous studies, since there is no firm consensus on the best preservation method, it is necessary to establish a protocol in which PRP is not affected by freezing in order to preserve its biological properties [20]. Furthermore, the effect of freezing on platelet activation and morphology has scarcely been studied. Therefore, the aim of this study is to analyze the effect that freezing for 6 and 12 months has on PRP, evaluating platelet morphology, GF levels, activation, and bioactivity on cell cultures.

## 2. Results

### 2.1. Platelet-Rich Plasma Characterization

The PRP used for this cryopreservation study had a platelet concentration of 403 ± 163 × 10^3^/µL and a higher platelet concentration than that of blood, at an average ratio of 2:4. It had no erythrocytes or leucocytes, and CaCl_2_ was used for activation (Table 1). In accordance with the UCS (Universal Coding System) and minimum reporting requirements for PRP studies, the PRP type was 14-00-11 [21].

### 2.2. Effect of Freezing on Platelet Concentration, Size and Fibrinogen Levels

**Figure 1:**
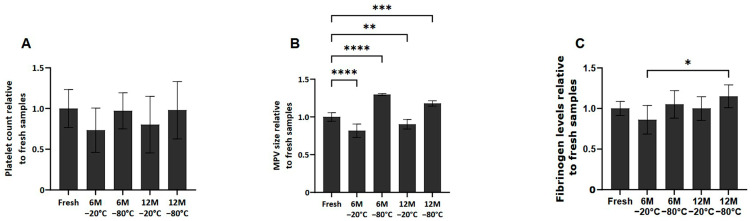
Platelet concentration, size and fibrinogen levels in fresh and cryopreserve samples. Mean values of platelet concentration (**A**), size (**B**) and fibrinogen levels (**C**) in the PRP by storage temperature (−20/−80 °C) and time (Fresh, 6 months, 12 months). Error bars = standard deviation (*n* = 6). Statistically significant differences were calculated using one-way ANOVA test (* *p* < 0.05; ** *p* < 0.01; *** *p* < 0.001; **** *p* < 0.0001).

No significant differences in platelet counts were observed among the different conditions after thawing the samples (Figure 1A). However, in terms of mean platelet volume (MPV), platelets frozen at −80 °C at 6 and 12 months showed an increase in size compared to fresh samples (*p* < 0.0001 and *p* < 0.001, respectively). However, the samples frozen at −20 °C at 6 and 12 months showed a decrease in MPV compared to fresh samples (*p* < 0.0001 and *p* < 0.01, respectively) (Figure 1B).

Regarding fibrinogen levels present in the fresh and cryopreserved samples (Figure 1C), although fibrinogen levels in the frozen samples are similar to those found in fresh samples, it has been observed that between samples frozen at 6 months −20 °C and 12 months −80 °C, there are statistically significant differences (*p* < 0.05).

### 2.3. Freezing Effect on Platelet Activation

**Figure 2:**
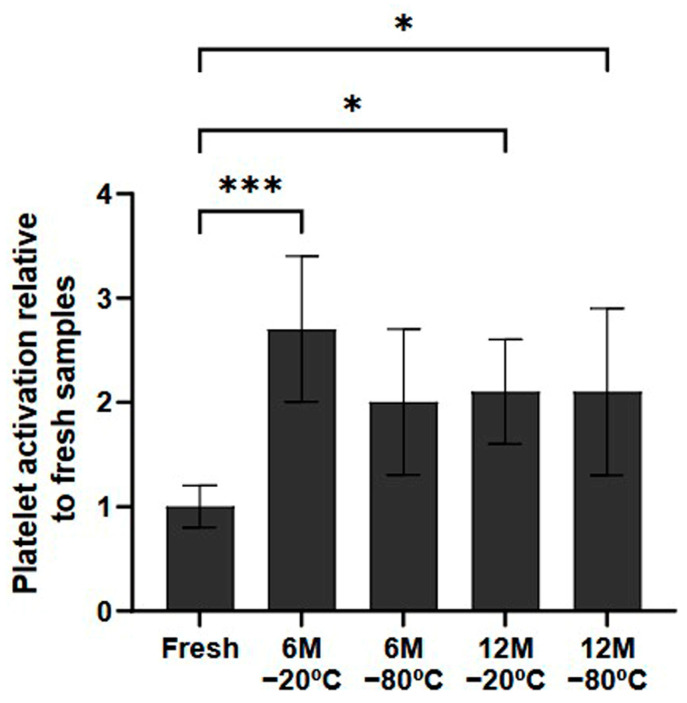
Platelet activation ratio after freezing preservation. Mean values of platelet expressing P-selectin in the PRP by storage temperature (−20/−80 °C) and time (Fresh, 6 months, 12 months). The values are shown in relation to the fresh sample. Error bars = standard deviation (*n* = 6). Statistically significant differences were calculated using a one-way ANOVA test (* *p* < 0.05; *** *p* < 0.001).

In terms of the potential for platelet activation during the freezing process, the samples that had been frozen indeed showed higher levels of platelet activation than fresh samples (Figure 2). These differences were statistically significant in all conditions stored for 12 months (*p* < 0.5, both). Regarding the samples stored for 6 months, although there was a general tendency towards higher platelet activation, only the samples stored at −20 °C showed significant differences (*p* < 0.001).

### 2.4. Growth Factor Profile After Cryopreservation

**Figure 3:**
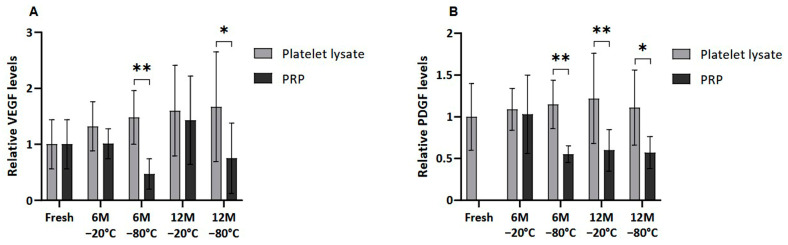
Platelet-derived GF levels in PL and PRP. Mean values of (**A**) VEGF levels and (**B**) PDGF-AB levels in plasma samples acquired in fresh and after 6 or 12 months frozen at −20 °C or −80 °C. Error bars = standard deviation (*n* = 6). Statistically significant differences were found using multiple Student’s *t*-tests (* *p* < 0.05; ** *p* < 0.01).

Several GFs, of both platelet and extraplatelet origin, were measured by ELISA in frozen PL and PRP in order to determine whether the freezing process could affect their release and/or degradation. VEGF and PDGF-AB, which are platelet-derived molecules, showed a similar pattern, where PRP freezing resulted in decreased levels of those factors in all the conditions (Figure 3). VEGF was significantly lower in PRP than in PL frozen at −80 °C for a period of 6 and 12 months (*p* < 0.01 and *p* < 0.05, respectively). In fact, significant differences were also found in PDGF-AB levels between samples frozen at −80 °C, both at 6 (*p* < 0.01) and 12 (*p* < 0.5) months. Moreover, PDGF-AB levels in PL were significantly higher than in PRP frozen at −20 °C for 12 months (*p* < 0.01).

Moreover, no significant differences were found between platelet-derived GF levels in fresh and both cryopreserved plasma conditions due the high variability of samples. Nevertheless, a clear trend of decreasing VEGF was observed in PRP frozen at −80 °C for 6 and 12 months compared to fresh samples. The same trend was observed in the case of PDGF-AB in all cases, except for the sample stored at −20 °C for 6 months.

**Figure 4:**
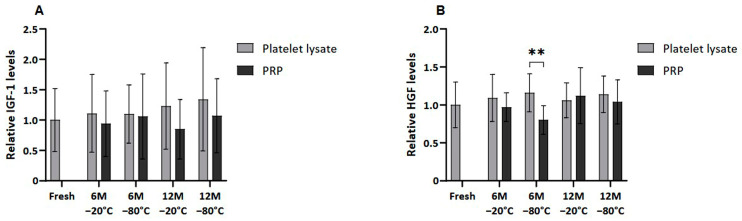
Extrapletelet GF levels in PL and PRP. Mean values of (**A**) IGF-1 levels and (**B**) HGF levels in plasma samples acquired in fresh and after 6 or 12 months frozen at −20 °C or −80 °C. Error bars = standard deviation (*n* = 6). Statistically significant differences were found using multiple Student’s *t*-tests (** *p* < 0.01).

Meanwhile, when extraplatelet GFs were analyzed (Figure 4), as is the case of IGF-1 and HGF, these differences were not observed except when freezing PRP at −80 °C for 6 months, which showed statistically significantly reduced HGF levels (*p* < 0.01).

### 2.5. Study of Platelet Functionality After Platelet Activation upon Cryopreservation

**Figure 5:**
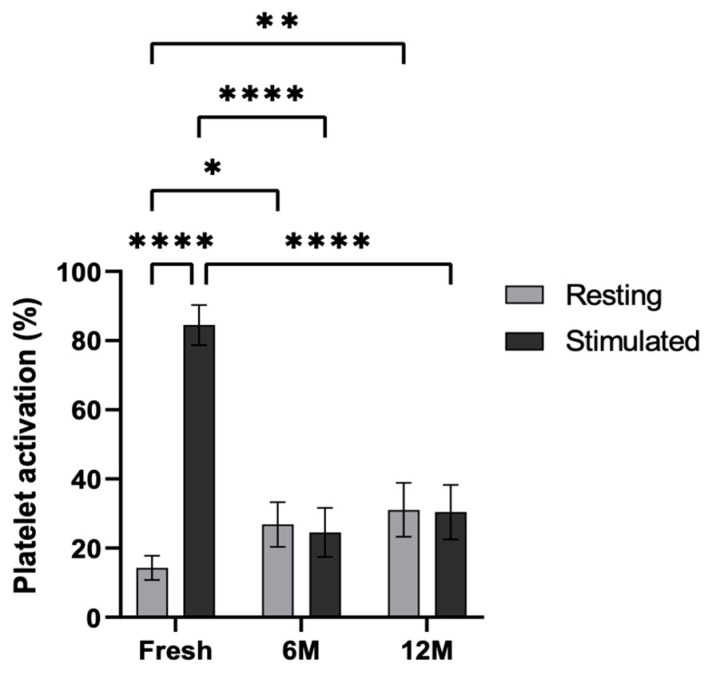
Platelet activation levels in resting and ADP-stimulated conditions. P-selectin (CD62p) expression was measured by flow cytometry in resting and ADP-stimulated platelets, both fresh, and following storage at −80 °C for 6 and 12 months. Error bars = standard deviation (*n* = 6). Statistically significant differences were calculated using a two-way ANOVA test (* *p* < 0.05; ** *p* < 0.01; **** *p* < 0.0001).

Cytometric analysis was carried out in order to study the functionality of the platelets after cryopreservation (Figure 5). To this end, the platelets were stimulated with adenosine diphosphate (ADP), and the P-selectin levels were measured in the fresh PRP as well as in the PRP frozen at −80 °C for 6 and 12 months. Platelet activation was statistically significant in the fresh condition following stimulation (*p* < 0.0001). Furthermore, in resting platelets, activation was significantly greater at both 6 and 12 months when frozen at −80 °C, compared to the fresh condition (*p* < 0.5 and *p* < 0.01). In contrast, lower activation was observed in ADP-activated platelets in platelets frozen at −80 °C for 6 and 12 months than in those under fresh conditions (*p* < 0.0001 and *p* < 0.0001, respectively). Nevertheless, no significant differences were found between resting and stimulated conditions at 6 months or 12 months.

### 2.6. Platelet Morphology After Platelet Freezing

**Figure 6:**
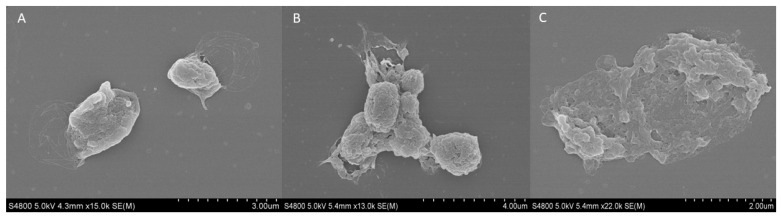
SEM analysis of external platelet morphology. Images of (**A**) fresh platelet, and platelets cryopreserved at −80 °C for (**B**) 6 months and (**C**) 12 months.

The external structure of fresh and cryopreserved platelets at −20 and −80 °C was analyzed using the scanning electron microscopy (SEM). The fresh platelets showed a spherical structure with a few small pseudopodia extending from the body with a fairly smooth surface and no platelet interaction (Figure 6A). Regarding the platelets frozen at −20 °C, an increase in surface roughness and platelet aggregation was observed (Figure 6B). Finally, when cryopreserving platelets at −80 °C, their spherical morphology and the integrity of the membrane were lost, and various porous structures appeared (Figure 6C).

### 2.7. Cellular Bioactivity of Cryopreserved Platelet-Rich Plasma

**Figure 7:**
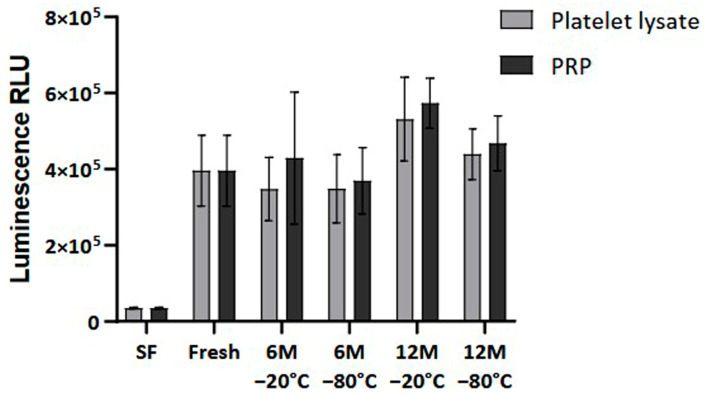
Cellular viability in Normal Human Dermal Fibroblast (NHDF) cells. The viability levels are expressed as relative light units (RLUs). NHDF cells were cultured in a medium supplemented with either fresh or frozen PL or PRP in triplicate, and cell viability was measured every 24 h. The serum-free (SF) condition served as negative control. Error bars = standard deviation (*n* = 6). Statistical analysis was calculated using a two-way ANOVA test.

Finally, cellular viability was measured to analyze the impact of the freezing process on the bioactivity of the PRP. The results showed no statistically significant differences in the viability and proliferation of the dermal fibroblasts at 96 h when comparing fresh and frozen conditions (Figure 7).

## 3. Discussion

The results of this study underscore the potential of long-term cryopreservation as a possible viable method for maintaining the properties and functionality of PRP. As the role of PRP in regenerative medicine continues to grow, understanding what impact certain preservation methods can have on its biological activity is essential. Our findings demonstrate that, although PRP preserved at −20 °C and −80 °C for up to 12 months could affect the morphology and functionality of platelets, it retains adequate cell viability, suggesting its utility in clinical settings.

In terms of platelet characteristics, freezing does not change the number of platelets, though preservation at −20 °C and −80 °C resulted in variations in platelet size. As previously noted by Beitia et al. [16], in the PRP samples preserved for 1 and 3 months at −20 °C, the platelet size increased, whereas there was no variation at −80 °C. In the present work, the platelets showed a significant decrease in size at 6 months, followed by an increase at 12 months. These variations may be the result of processes that alter the morphology and functionality of platelets, or even the result of platelet aggregation [22]. Indeed, research indicates that platelet size affects their response to cryopreservation [23]. Larger platelets tend to preserve their responsiveness better than smaller platelets [24,25], which is evident in their higher aggregation rates post-cryopreservation. However, larger platelets also experience a greater loss of secretion granules compared to smaller ones, suggesting that different size fractions of platelets have varying vulnerabilities to cryopreservation-induced damage [24].

Regarding fibrinogen levels in cryopreserved PRP, although no change in fibrinogen levels was observed in most of the conditions, there was a slight but significant increase between 6 months at −20 °C and 12 months at −80 °C. This could be because storing platelets at −80 °C enhances their procoagulant properties, leading to earlier thrombin and fibrin formation. However, this may be clinically irrelevant, as cryopreserved platelets are effective in reducing blood loss and improving hemostatic function, with an associated increase in fibrinogen levels [26]. Furthermore, it is important to mention that in all conditions the fibrinogen concentration did not exceed the maximum values allowed in humans, being the range 150–400 mg/dL [27]. These findings support the potential benefits of cryopreserved platelets in emergency and surgical settings.

In order to analyze platelet functionality, a platelet activation assay was performed to detect the expression of P-selectin, an indicator of activated platelets. The results showed an increase in platelet activation when the platelets were cryopreserved. As mentioned before, platelet MPV increases in response to platelet activation, which can be triggered by various stimuli, such as collagen and temperature changes. For instance, exposure to low temperatures increases MPV, while higher temperatures decrease it, indicating that changes in MPV may favor platelet activation under different conditions [28]. Moreover, it has been demonstrated that high temperature changes result in platelet activation, which is why freeze/thaw cycles are commonly used for platelet activation [29,30].

The effect of cryopreservation on GF levels in PRP and PL was analyzed. The study highlights a notable reduction in critical GFs, such as PDGF-AB and VEGF, following cryopreservation. In the present study, no significant reduction in GFs was found in the frozen samples compared to fresh controls, as reported by Hosnuter et al. and Beitia et al. [16,31]. Nevertheless, frozen PRP showed a clear trend of lower PDGF-AB and VEGF than in fresh samples, especially at lower temperatures and for longer cryopreservation periods. Moreover, PDGF-AB and VEGF levels were significantly lower in PRP stored at −80 °C for 6 and 12 months compared to PL. PDGF-AB levels were also lowered in PRP frozen at −20 °C for 12 months. These results coincide with the previous study by Beitia et al., which states that the preservation of PL is more favorable to that of PRP [16]. Regarding extraplatelet GFs, the levels remained unchanged in all groups. Moreover, no significant differences between fresh and frozen samples were seen. Only slightly lower amounts of HGF were observed in PRP than in PL when frozen at −80 °C for 6 months. It should be noted that HGF is both a platelet and an extraplatelet GF [9], so this difference could be due to platelet alteration.

The maintenance of extraplatelet GF levels ensures the bioactivity of PRP, as both platelet and extraplatelet GFs have been shown to play a significant role in supporting PRP bioactivity, particularly IGF-1 [9,32,33,34]. In fact, these results are in agreement with those obtained for bioactivity, since cell proliferation was not altered between the frozen and fresh samples, and the bioactivity of frozen PRP was maintained over long periods both at −20 °C and −80 °C. In addition, it is important to mention the role of exosomes in tissue repair. It is possible that, after freezing, they are still viable and are contributing to cell proliferation [35,36].

The decrease in platelet factor levels in PRP could be a consequence of structural changes caused by cryopreservation, affecting the functionality of the PRP. Platelets thawed after preservation at −80 °C showed twice as much platelet activation as a fresh sample. After adding ADP to PRP, neither the stimulated nor the unstimulated samples showed increased activation, unlike fresh plasma, where platelets became activated upon stimulation. This assay indicates that, after freezing, platelets are no longer functional due to platelet activation, and they do not respond following the addition of an activator such as ADP or CaCl_2_, inhibiting their activation and preventing the release of biomolecules such as GFs.

In relation to this, the morphological study of the platelet in samples frozen at −80 °C showed increased deterioration of the platelet membrane at 6 and 12 months. This deterioration is visible, as the outer membrane is wrinkled compared to the fresh platelet, whose round morphology and intact membrane can be observed. In addition, the open canalicular system (OCS) in the membranes of the frozen platelets can be observed at both time points. The OCS is a complex network of channels that plays a crucial role in platelet activation and the release of GFs. This system facilitates the transport of substances into platelets and the discharge of alpha granule products during the platelet release reaction [37]. The OCS may collapse or evaginate onto the platelet surface, indicating a dynamic structural change in response to activation, inhibiting the release of alpha-granule content [38]. Moreover, at both 6 and 12 months, platelet aggregation was observed, which could be related to the increase in size affecting platelets frozen at −80 °C, as has been described in a previous study [22].

These findings highlight the potential of cryopreserved PRP as a practical alternative in clinical applications. Although studies indicate that PL could be more suitable for preservation than PRP, the latter preserves its bioactivity such that it can be applied when a controlled release system of GF is desired. This is achieved by activating the PRP prior to injection, triggering coagulation and generating the fibrin matrix where GFs are anchored and gradually released over time [39]. Long-term storage could allow for more flexible treatment schedules and support the development of PRP biobanks. In addition, it would enable easier access to high-quality PRP samples, which could expand regenerative treatment options, optimize outcomes, and promote personalized medicine. Meanwhile, future studies should continue refining cryopreservation protocols to maximize both the structural integrity and biological activity of PRP, further solidifying its role in regenerative medicine.

The main limitation of this work is that further in vitro assays could have been carried out, as only cell viability has been examined, in addition to sample size. In fact, it could be interesting to analyze cell migration, inflammation, apoptosis and oxidative stress in an in vitro and in vivo environment. Moreover, it would be interesting to analyze cell bioactivity in other cell types such as chondrocytes and osteoblasts, among others. In addition, to better understand what happens to the platelet in terms of its morphology and content, it would be useful to carry out a Transmission Electron Microscopy (TEM) analysis and a more in-depth study on how platelet exosomes are released from the platelet and whether they are functional after freezing. Finally, new and future studies, such as the analysis of platelet aggregation and adhesion as well as the observation of chemical and signaling changes, like P-selectin shedding in the platelet membrane, could provide further answers on the effect of long-term cryopreservation.

## 4. Materials and Methods

### 4.1. Donors

Blood was obtained from six healthy individuals (3 females/3 males), whose ages ranged between 25 and 60. Each donor signed the informed consent. The ethical approval for the study was obtained from the Ethics Committee of OSI Araba (Protocol No. UCA-14/EE/22/CON).

### 4.2. Platelet-Rich Plasma and Platelet Lysate Obtaining Procedure

Blood was collected from donors in 9 mL tubes containing 3.8% (*w*/*v*) sodium citrate. Blood fragmentation was performed by centrifugation at 580× *g* for 8 min at room temperature. To obtain the PRP, the plasma fraction corresponding to the upper yellowish layer was collected, excluding the intermediate leukocyte layer and the erythrocyte layer at the bottom. To obtain the PL, half of the PRP volume was separated and CaCl_2_ (10% *w*/*v*) was added to trigger the platelet activation cascade. After incubation at 37 °C for approximately 30 min, the generated clot was squeezed and discarded, reserving only the remaining volume.

### 4.3. Platelet-Rich Plasma and Platelet Lysate Preservation

PRP and PL were obtained at different time points. The first extraction was performed 12 months before the analysis, the second one 6 months before, and the third one the same day of the analysis in order to be considered a fresh control. Thus, after each extraction, both PRP and PL were stored in aliquots at −20 °C and −80 °C. Both temperatures were selected because they are the two most commonly used for preserving biological samples. On the day of obtaining the fresh samples and, therefore, that of the experimental activity, CaCl_2_ (10% *w*/*v*) was added to all PRP samples (fresh and cryopreserved at −20 °C and −80 °C). After clot formation, it was squeezed and the remaining volume was reserved. This allowed comparisons to be made between the frozen PRP and PL as well as between different storage temperatures.

### 4.4. Measurement of Platelet Number, Size and Fibrinogen Levels

The platelet concentration levels and size were measured by means of the hematological analyzer Sysmex XS-1000i (Sysmex, Kobe, Japan). The MPV was measured to determine the platelet size. Based on these data, the PRP was classified according to the UCS (universal coding system) [21]. For the analysis of the fibrinogen levels, 400 µL of plasma volume was collected from all conditions and measured using the coagulation analyzer (STA Compact Max, Stago, France).

### 4.5. Platelet Activation Capacity Measurement

Flow cytometry was used to assess cryopreservation-mediated platelet activation, analyzing platelet labeling and activated platelet labeling. To this end, the following fluorochrome (FITC or PE) conjugated monoclonal antibodies were used: anti-CD41-FITC and anti-CD62P-PE (BD Biosciences, San Jose, CA, USA). For each donor, 10 μL of plasma sample, 5 μL of anti-CD41-FITC and 5 μL of anti-CD62P-PE were added to the test tubes. The remaining volume was brought up to 100 μL with PBS. In addition, to determine whether previously cryopreserved platelets maintain their activation capacity, 11 μL of ADP at 50 μM, diluted in PBS, was added to the final solution. Appropriate controls for the flow cytometric technique were used. Finally, the samples were incubated for 15 minutes at room temperature in the dark, then fixed with 400 μL of freshly prepared 1.25% formaldehyde in PBS. The samples were stored at 4 °C in darkness until their analysis in 24 h. A Gallios flow cytometer (Beckman–Coulter, High Wycombe, UK) was used to analyze the events.

### 4.6. Platelet Morphology Study

The platelet external structure was analyzed using SEM (JSM-7000F, JEOL, Japan). Briefly, 20 μL of PRP were added to a glass coverslip and placed onto filter paper dampened with PBS at 37 °C for 5 min. After that, the samples were rinsed in PBS for 20 min on the benchtop without shaking to avoid platelet loss. Once the excess of plasma and plasmatic proteins were removed, the samples were fixed in 2.5% glutaraldehyde solution for 30 min. Next, three washes were performed in PBS for 5 min and the samples were fixed with 1% osmium tetra-oxide (OsO_4_) for 15 min, followed by a second rinsing process, as described above. Finally, the samples were dehydrated with serial concentrations of ethanol (30, 50, 70, 90 and three-fold 100%) for 5 min each. The samples were critical point dried (Tousimis Int. J. Mol. Sci. 2024, 25, 4069 10 of 13 Autosamdri 814; Tousimis, Rockville, MD, USA), sputter-coated with 5 nm gold (Edwards E306A; Edwards Vacuum, Burgess Hill, UK), and subsequently examined under a scanning electron microscope (Hitachi S-4800; Hitachi, Tokyo, Japan) at the UPV/EHU.

### 4.7. Measurement of Growth Factor Levels

The GF levels were measured using specific ELISA kits for each protein (Bio-techne; Minneapolis, MN, USA), following the manufacturer’s instructions. PL and PRP samples were measured. The PL samples correspond to plasma previously activated with CaCl_2_ and subsequently frozen, while the PRP samples correspond to unactivated frozen plasma, keeping the platelets intact. After thawing both samples, the PRP samples were activated with CaCl_2_ for the release of GFs. The GFs tested were Hepatocyte Growth Factor (HGF) (156–10,000 pg/mL), Insulin-Like Growth Factor 1 (IGF-1) (94–600 ng/mL), Platelet-Derived Growth Factor (PDGF-AB) (780–50,000 pg/mL) and Vascular Endothelial Growth Factor (VEGF) (31.3–2000 pg/mL). Absorbance was measured using the Tecan Infinite F200 PRO (Tecan; Männedorf, Switzerland), where the concentrations of each GF were extrapolated based on the calibration curve obtained. The samples were analyzed in duplicate.

### 4.8. Cell Culture

NHDF (CC-2511; Lonza, Basel, Switzerland) were used for the in vitro analysis. They were incubated at 37 °C and 5% CO_2_ in fibroblast basal medium (CC-3131; Lonza, Basel, Switzerland) supplemented with insulin, Human Fibroblast Growth Factor (FGF) and gentamicin sulfate-amphotericin at 0.1% (*v*/*v*) each (CC-4126; Lonza, Basel, Switzerland), as recommended by the manufacturer. Additionally, CaCl_2_-activated PRP and PL, which had been frozen at −20 °C or −80 °C for either 6 or 12 months, were added to the media at a concentration of 10%. For the negative control, no serum was added.

### 4.9. Cell Viability Assay

To assess whether the bioactivity of PRPs could be affected by long-term storage temperature, dermal fibroblast cells incubated with medium supplemented with PRP stored at different temperatures were cultured in triplicate with each of the pools. Cell viability assay was performed at 96 h using the luminescence-based Realtime-Glo MT Cell Viability Assay kit (G9711; Promega, Fitchburg, WI, USA). The signal was measured using a Tecan Infinite F200 PRO (Tecan; Männedorf, Switzerland). The levels of luminescence that the detector reached could be considered proportional to the number of viable cells present in the assay. The obtained cell GFs with the different PRP formulations were compared in order to assess whether cryopreservation affects the bioactivity of PRP.

### 4.10. Statistical Analysis

The comparisons were performed using one-way and two-way ANOVA (analysis of variance), followed by a Tukey/Dunnett multiple comparison test and multiple Student’s *t*-tests. The normal distribution of samples was assessed using the Shapiro–Wilk test. The data were considered statistically significant when the *p*-values were less than 0.05. The statistical analysis was performed with PASW Statistics 20.0 (SPSS^®^, Chicago, IL, USA) and GraphPad Prism (San Diego, CA, USA).

## 5. Conclusions

In conclusion, this study supports the feasibility of the long-term cryopreservation of PRP at −20 °C and −80 °C, demonstrating that PRP can retain its key properties and bioactivity even after 6 to 12 months of storage. Although cryopreservation led to variations in platelet size and morphology as well as increased activation levels, the PRP’s general bioactivity remained intact. Although a reduction in platelet-derived GFs was observed, the levels of extraplatelet GFs remained stable, which helps maintain the PRP’s therapeutic effectiveness.

## Figures and Tables

**Table 1:** Summary of PRP characteristics.

**1. PRP Preparation**	
Initial blood volume	9 mL
Anticoagulant	Sodium citrate 3.8% (*w*/*v*)
System	Close
Centrifugation	Yes
number	1
speed	580× *g*—8 min
Final PRP volume	4 mL per subject
**2. PRP Characteristics**	
PRP Type	14-00-11
MPV	10 ± 1 fL
Red Blood Cells	<0.01 × 10^6^/µL
White Blood Cells	<0.05 × 10^6^/µL
Neutrophils	---
Lymphocytes	---
Monocytes	---
Eosinophils	---
Basophils	---
Activation	CaCl_2_ (10% *w*/*v*)
**3. Application Characteristics**	
Dose	10%
Direct/Indirect	Direct
Cell line	Normal Human Dermal Fibroblast
**4. Other remarkable PRP and study features**	
The product added to the cell cultures was the PL obtained after activation of PRP using calcium chloride (10%)	

## Data Availability

The original contributions presented in this study are included in the article. Further inquiries can be directed to the corresponding author.
